# Supplementary material for: BRCA2 deficiency and replication stress drive APOBEC3-Mediated genomic instability
Source: Nat Commun. 2025 Oct 29;16:9544. doi: 10.1038/s41467-025-64578-6 (PMC12572151; doi:10.1038/s41467-025-64578-6)
Supplement: Supplementary file 1 — Supplementary Information [file 41467_2025_64578_MOESM1_ESM.pdf]

Supplementary Figures and Tables to:

BRCA2 Deficiency and Replication Stress Drive APOBEC3-Mediated Genomic Instability

Kathy Situ [#,1], Haohui Duan[#,1], Stephen K Godin[1], Joshua Yang[1], Gabrielle Q McCloskey[1], Basim Naeem[1], Margaret K Gillis[1], Muhammad H Zeb[1], Silvi Salhotra[1], Pratha Rawal[1], Nisha Patel[1], Salome K Mouliere[1], Jie Chen[2], Angéla Békési[3,4], Hajnalka L Pálinkás,[3,4], Subramanian Venkatesan[5], Abby M Green[6], Nicolai J Birkbak[7,8], Beáta G Vértessy[3,4], Charles Swanton[5,9,10], Shailja Pathania[2]*

Supplementary Figure 1

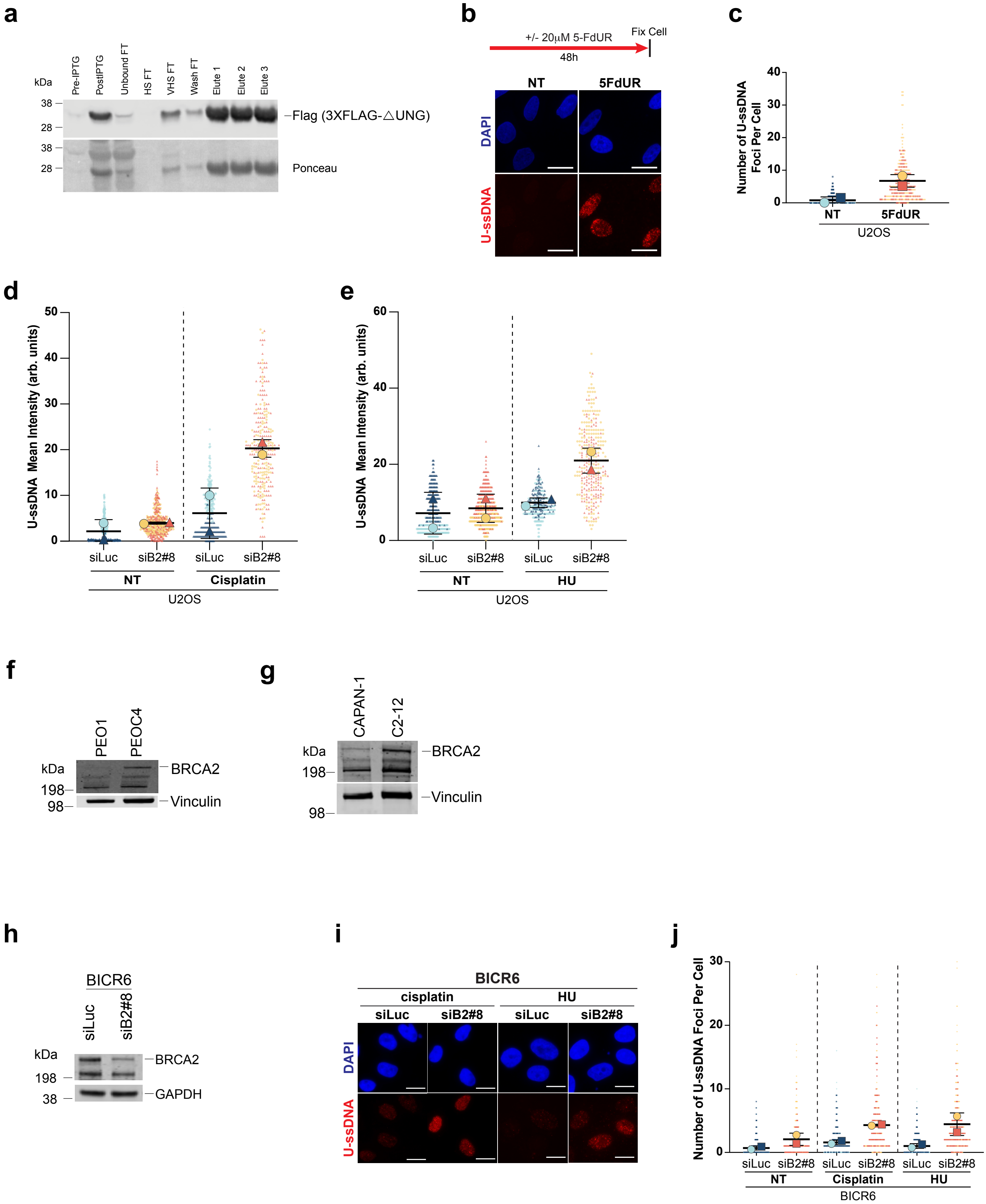

Supplementary Fig. 1: BRCA2 deficient cells accumulate U-ssDNA upon replication stress. **(a)** Western blot showing purification of the 3xFLAG-△UNG probe. Top: anti-FLAG for 3xFLAG-△UNG detection; bottom: Ponceau stain for total protein. FT: Flowthrough; HS: High salt, VHS: Very high salt. **(b)** (Top) Schematic of 5-FdUR treatment. (Bottom) Representative images of U-DNA foci detected with 3xFLAG-△UNG probe in untreated (NT) or 20µM 5FdUR-treated cells (48h). **(c)** Quantification of U-ssDNA foci from (b). SuperPlots of n=2 replicates are plotted. U-ssDNA foci/cell were quantified using Image J (100-200 cells/replicate). Each highlighted shape (circle and square) represents the average of each replicate, with the black lines representing the mean ± SD of n=2 independent experiments. (**d-e**) Quantification of U-ssDNA foci in U2OS cells transfected with indicated siRNAs and treated with (d) cisplatin (50µM, 1h, 24h recovery) or (e) HU (2mM, 30h, no recovery). SuperPlots of two independent experiments are shown. U-ssDNA foci per cell was analyzed as described in (c). **(f-g)** Western blot of BRCA2 in (f) PEO1 and PEOC4, and (g) CAPAN-1 and C2-12 tumor cells. Vinculin is the loading control. **(h)** Western blot of BRCA2 in BICR6 cells transfected with the indicated siRNAs (48h). GAPDH is the loading control. **(i)** Representative images of U-ssDNA foci in BICR6 cells transfected with the indicated siRNAs for 48h, followed by cisplatin or HU treatment. **(j)** Quantification of U-ssDNA foci for experiment described in (i). SuperPlots of 2 independent experiments (n=2) are shown and quantified as in (c). Scale bar represents 20µm in the IF images shown here. Western blot images presented here are representative of three or more western blots with similar results. Source data are provided as a Source Data file.

Supplementary Figure 2

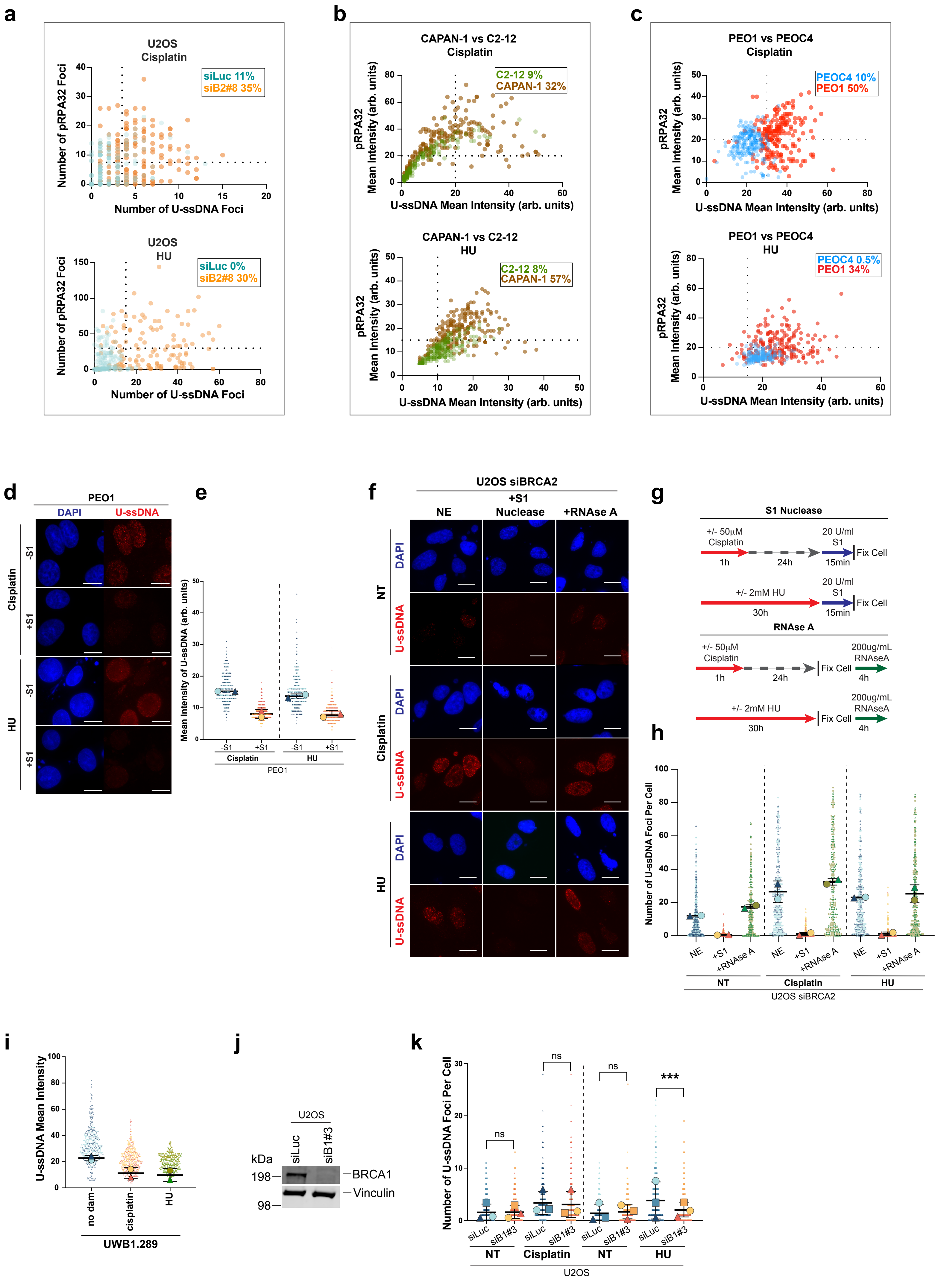

Supplementary Fig. 2: Replication stress-induced ssDNA in BRCA2- but not BRCA1-deficient cells is prone to uracil accumulation.

(a) Quantification of U-ssDNA and pRPA32 foci per cell in siLuc and siBRCA2 cells transfected with cisplatin or HU. Dashed lines indicate the baseline foci count; cells above baseline are used to calculate percentages. **(b - c)** Quantification of U-ssDNA and pRPA32 foci mean intensity in (b) CAPAN-1/C2-12 and (c) PEO1/PEOC4 cells treated with cisplatin and HU as described in (a). **(d - e)** Quantification (d) of and representative images (e) of U-ssDNA accumulation in PEO1 cells treated with cisplatin or HU (2mM), with or without S1 nuclease. Cells were incubated with 20U/ml S1 nuclease at 37ºC for 15 min before fixation. U-ssDNA was detected using 3xFLAG-ΔUNG probe. SuperPlots of n=2 independent experiments are shown (100-200 cells/replicate). Each highlighted shape (circle, triangle or square) represents the average of each replicate, with black lines indicating mean ± SD. **(f – h)** (f) Representative images, (g) schematic of the treatment timeline (+/- cisplatin or HU followed by S1 nuclease (20 U/mL) or RNAseA treatment), and (h) quantification of U-ssDNA accumulation in U2OS cells transfected with the indicated siRNAs. S1 nuclease and RNase A treatment was done at 37C (details in Methods). SuperPlots of 2 independent experiments are shown (100-200 cells/replicate) (n=2). **(i)** Quantification of U-ssDNA mean intensity in UWB1.289 *BRCA1*-mutant ovarian cancer line treated with cisplatin or HU. SuperPlots of n=2 are shown and analyzed as described in (e). **(j)** Western blot of BRCA1 in U2OS cells transfected with BRCA1 siRNA (siB1#3). Vinculin is the loading control**. (k)** Quantification of U-ssDNA foci in U2OS cells transfected with the indicated siRNAs prior to treatment with cisplatin and HU. SuperPlots of 3 independent experiments (n=3) are shown per condition (100-200 cell/replicate) and analyzed as described in (e). Statistical significance for all the sets of three independent biological replicates (n=3) analyzed above was determined using repeated-measurement model followed by two-tailed multiple comparisons with Bonferroni post hoc test. ***$p \leq 0.001$; ns, not significant. In all images, scale bar represents 20 µm. Western blot images presented here are representative of three or more western blots with similar results. Source data are provided as a Source Data file.

Supplementary Figure 3

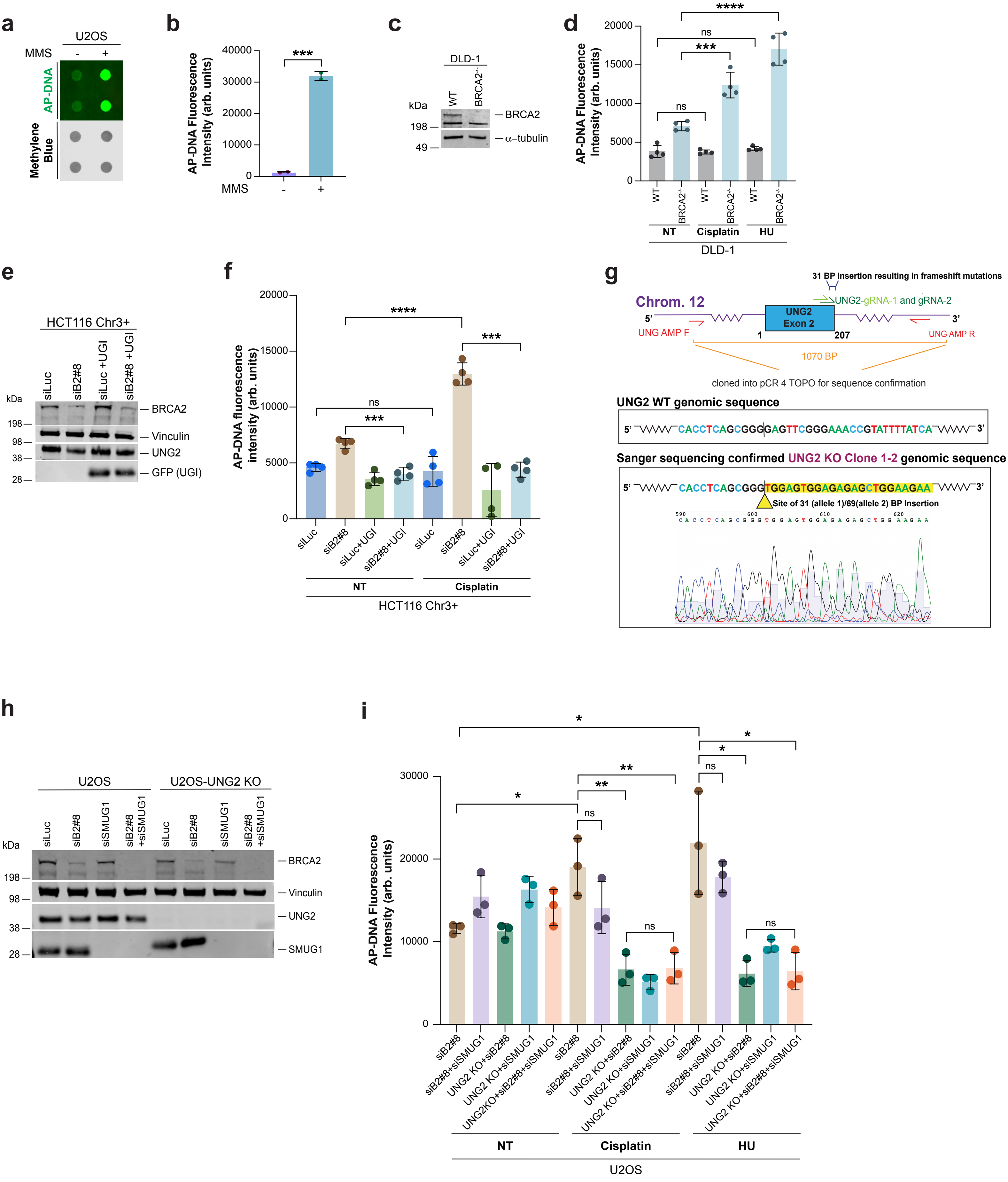

Supplementary Fig. 3: UNG2-driven uracil removal induces abasic site accumulation in BRCA2-deficient cells upon replication stress.

(a) Representative image of dot blot of AP-DNA in U2OS cells treated with 10mM MMS for 2hrs. Methylene blue is the loading control. One of two independent experiments is shown. **(b)** Quantification of dot blot in (a). Fluorescence intensity is plotted (arb. units). Data are mean ± SD of n=2 independent experiments. **(c)** Western blot of BRCA2 in DLD-1 WT and $BRCA2^{-/-}$ cells. α-tubulin is the loading control. **(d)** Quantification of AP-DNA fluorescence intensity normalized to loading control in DLD-1 cells treated with cisplatin (50µM, 1h; 24h recovery) or HU (2mM, 30h). Data are mean ± SD of n=4 independent experiments. Statistical significance was determined by un-paired two-tailed Student's *t* test. ****p≤0.0001; **p≤0.01; *p≤0.05. **(e)** Western blot of BRCA2 and UNG2 in HCT116 Chr3+ (+/-UGI). GFP confirms UGI expression. Vinculin is the loading control. **(f)** Quantification of AP-DNA fluorescence intensity normalized to loading control in HCT116 Chr3+ +/-UGI transfected with the indicated siRNAs and treated with cisplatin as described in (d). Data are mean ± SD of n=4 independent experiments. Statistical significance was determined as described for (d). ****p≤0.0001; ***p≤0.00; ns: not significant p>0.05. **(g)** Schematic of U2OS UNG2-KO sequence. A double nickase plasmid containing UNG2-gRNA-1 and gRNA-2 was transfected into U2OS cells. Sequencing revealed 31bp (allele 1) and 69bp (allele 2) insertions resulting in frameshifts, as shown in chromatogram. **(h)** Western blot of BRCA2, UNG2, and SMUG1 in and UNG2-KO U2OS cells transfected with the indicated siRNAs. Vinculin is the loading control. **(i)** Quantification of AP-DNA fluorescence intensity normalized to loading control in U2OS or UNG2-KO U2OS cells transfected with the indicated siRNAs and treated with cisplatin and HU as described in (d). Data are mean ± SD of n=3 independent experiments. Statistical significance was determined as described in (d). **p≤0.01; *p≤0.05; ns: not significant p>0.05. Western blot images presented here are representative of three or more western blots with similar results. Source data are provided as a Source Data file.

Supplementary Figure 4

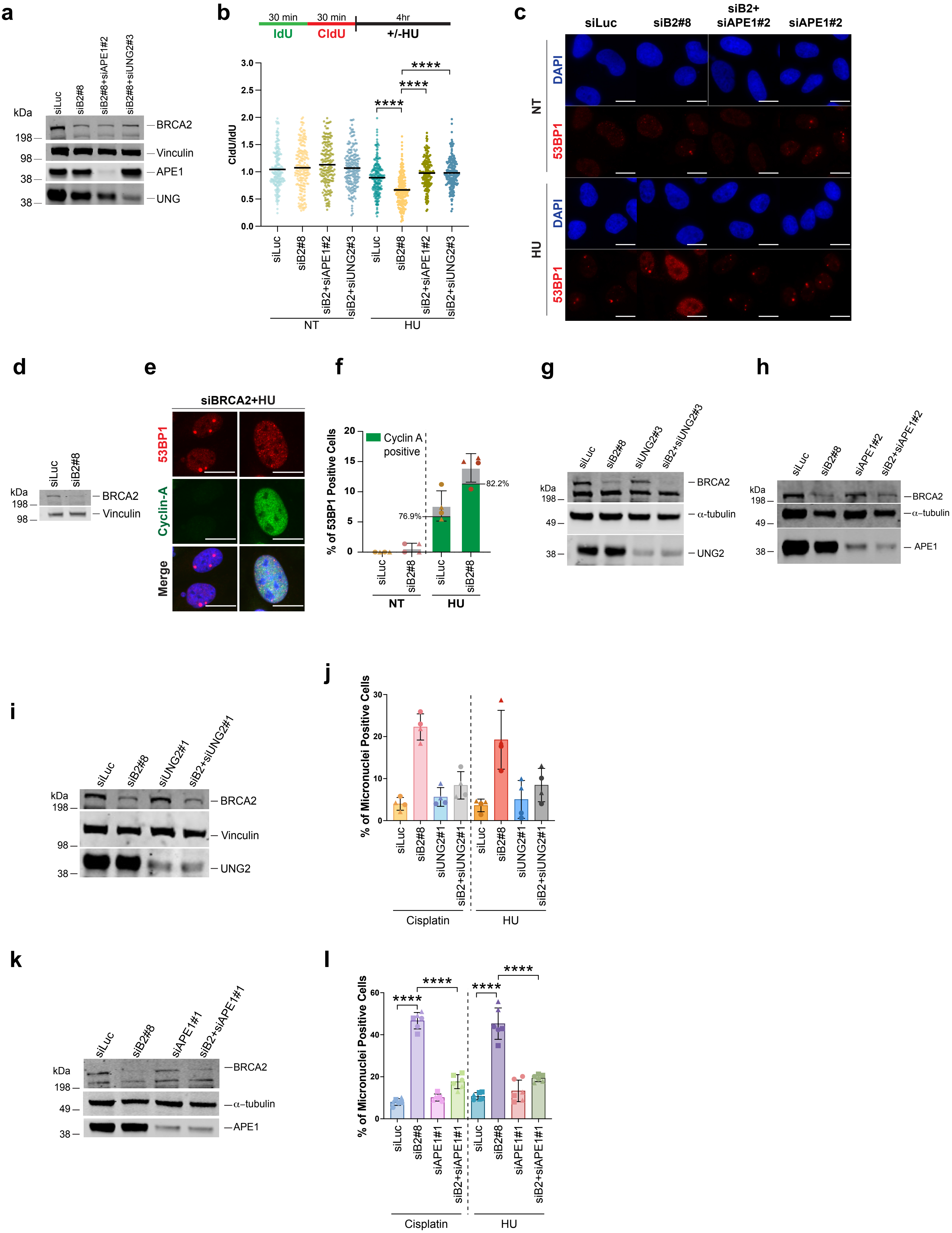

Supplementary Fig. 4: UNG2 and APE1 drive stalled fork degradation and genomic instability in BRCA2-deficient cells.

(a) Western blot of BRCA2, APE1, and UNG2 in U2OS cells transfected with the indicated siRNAs. Vinculin is the loading control. **(b)** Top: Labeling scheme for DNA fiber assay. Bottom: Quantification of CldU/IdU ratios in cells transfected with the indicated siRNAs and treated with and without HU (5mM, 4h). 150-200 fibers were analyzed per experiment. One of two independent experiments is shown. Statistical significance was determined by un-paired two-tailed Student's *t* test. ****p≤0.0001. A second biological replicate is presented in Supplementary Data 1. **(c)** Representative images of 53BP1 staining in U2OS cells transfected with the indicated siRNAs and treated with HU (5mM for 4h, 18h recovery). Scale bar represents 20μm. **(d)** Western blot of BRCA2 in U2OS cells transfected with the indicated siRNAs. **(e)** Representative images of 53BP1 and cyclin-A in siBRCA2 U2OS cells treated with HU (2mM, 30h). Scale bar represents 20μm. **(f)** Quantification of 53BP1 positive cells in (e). Green bars indicate the fraction also positive for cyclin-A. Each shape (circle or triangle) represents a biological replicate (n=2) with technical replicates (n=2, >100 cells/replicate). **(g, h)** Western blots of (d) BRCA2 and UNG2 or (e) BRCA2 and APE1 in U2OS cells transfected with indicated siRNAs. α-tubulin is the loading control. Knockdowns correspond to Fig. 4j-m. **(i, k)** Western blots of (i) BRCA2 and UNG2 or (k) BRCA2 and APE1 in U2OS cells. Vinculin and α-tubulin serve as the loading control. **(j, l)** Quantification of micronuclei-positive cells in U2OS cells and treated with cisplatin (50μM, 1h; 48h recovery) or HU (2mM, 30h; 24h recovery) (l). Data are mean ± SD of n=2 (j) or n=3 (l) independent experiments two technical replicates per experiment (150-200 cells/replicate). Statistical significance (l) was determined by un-paired two-tailed Student's *t* test. ****p≤0.0001. Western blot images presented here are representative of three or more western blots with similar results. Source data are provided as a Source Data file.

Supplementary Figure 5

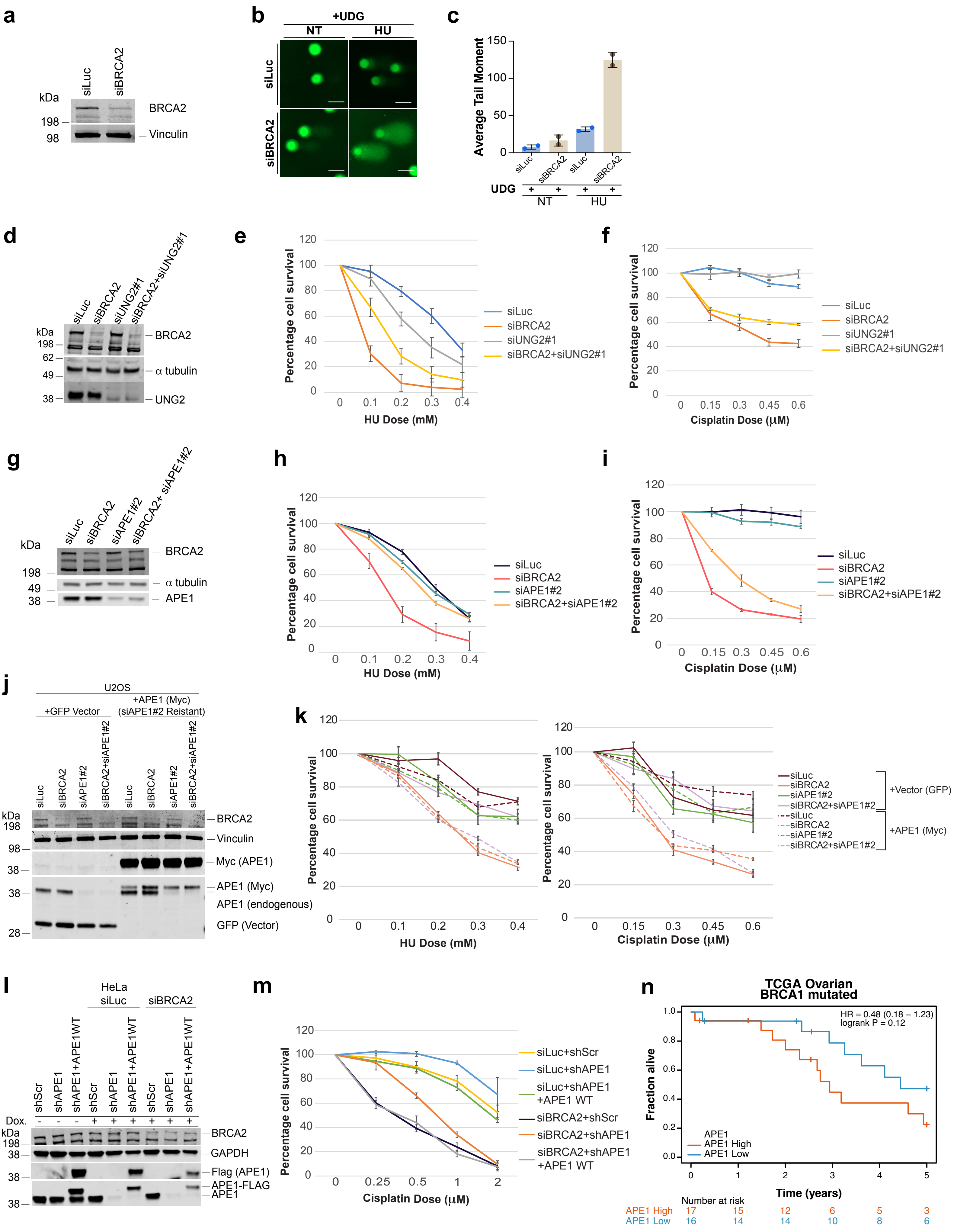

Supplementary Fig. 5: UNG2 and APE1 drive genomic instability in BRCA2-deficient cells. **(a)** Western blot of BRCA2 in U2OS cells transfected with siBRCA2. Vinculin is the loading control. **(b)** Representative images of UDG-modified alkaline comet assay in U2OS cells transfected with the indicated siRNAs. Cells were treated with cisplatin (50µM, 1h; 24h recovery) or HU (2mM, 30h). Agarose embedded cells were lysed and incubated with UDG (10U/ml, 1h, 37ºC) prior to electrophoresis. One of two independent experiments is shown. Scale bar represents 50µm. **(c)** Quantification of tail moment from (b). 100-150 comets/experiment were analyzed. Data are mean ± SD of n=2 independent experiments. **(d, g)** Western blots of BRCA2 with UNG2 (d) or APE1 (e) in U2OS cells transfected with indicated siRNAs. α-tubulin is the loading control. **(e-f, h-i)** CellTiter-Glo survival assays in cells treated with HU (e and h) or cisplatin (f and i) for 4 days, followed by 2 days of recovery. Error bars represent SD between triplicates. (**j)** Western blot of BRCA2, APE1, and Myc tagged APE1 in U2OS cells transfected with indicated siRNAs. Cells were also transfected with vector-GFP or siAPE1#2 siRNA resistant APE1-Myc. Vinculin is the loading control. **(k)** CellTiter-Glo assays for cells in (j) treated with cisplatin for 4 days followed by 2 days of recovery. Error bars represent SD between triplicates. (**l)** Western blot of BRCA2, APE1, and Flag -tagged APE1 in HeLa cells stably expressing doxycycline-inducible shAPE1 +/- stably expressing shRNA-resistant APE1. Vinculin is the loading control. **(m)** CellTiter- survival assays for HeLa cells in (l) treated with cisplatin as described in (k). **(n)** Kaplan-Meier curve of 5-year survival for patients in the TCGA ovarian cancer cohort, with BRCA1-deficient tumors. The patients are split by median expression of APE1. A list of mutations associated with the patient data used in this study are listed in the Source Data – Supplementary Figures. Western blot images presented here are representative of three or more western blots with similar results. Source data are provided as a Source Data file.

Supplementary Figure 6

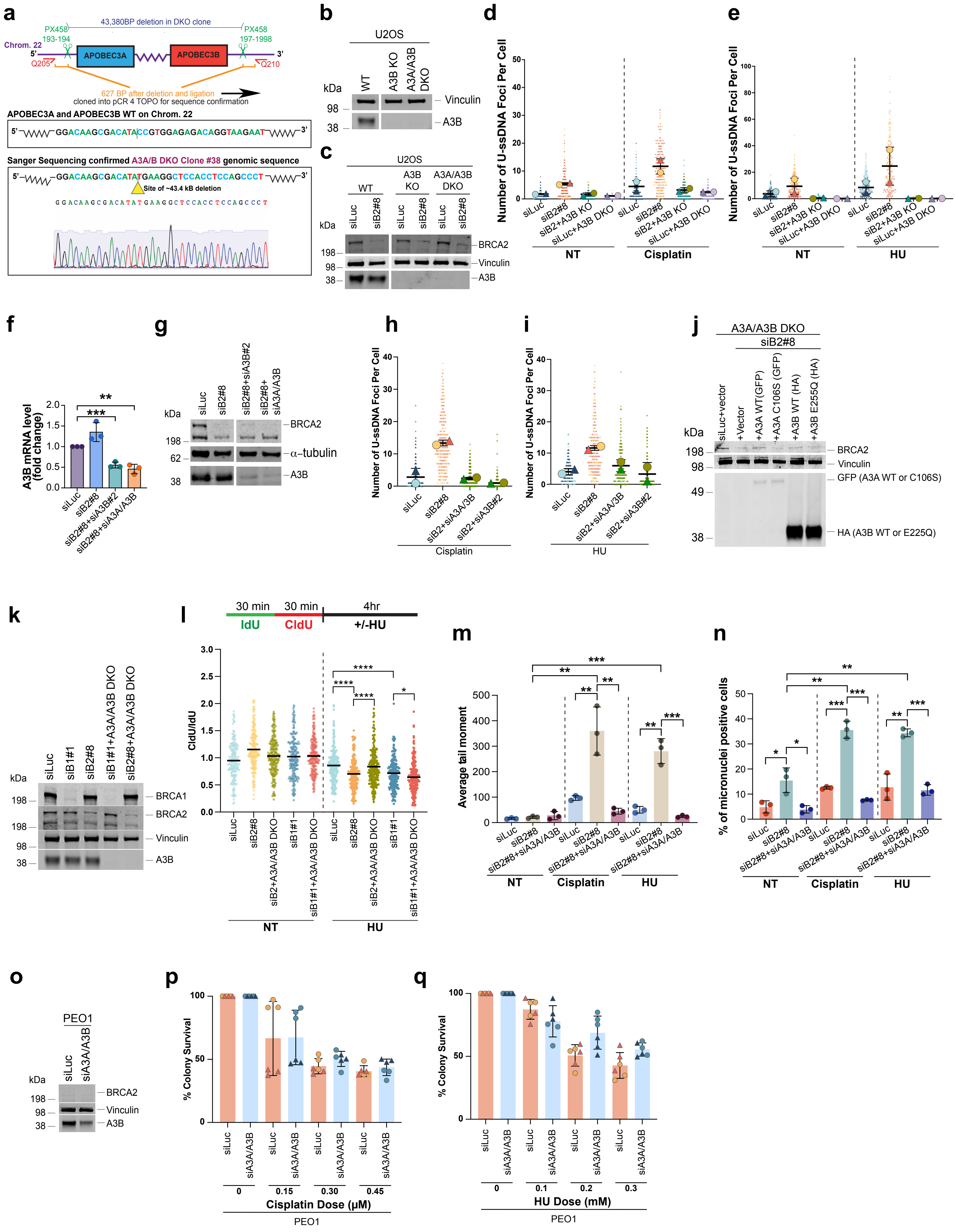

Supplementary Fig. 6: APOBEC-induced uracil accumulation drives genomic instability in BRCA2-deficient cells upon replication stress.
(a) Schematic of A3A/A3B double knockout (DKO) strategy in U2OS cells. Sequencing confirmed a 43.38kB deletion across the A3A and A3B. **(b)** Western blot of A3B in A3B KO or A3A/A3B DKO U2OS cells. Vinculin is the loading control. **(c)** Western blot of BRCA2 and A3B in A3B KO or A3A/A3B DKO U2OS cells transfected with indicated siRNAs. **(d, e)** Quantification of U-ssDNA foci in A3B WT or A3B-KO U2OS cells transfected with indicated siRNAs and treated with cisplatin (50µM, 1h; 24h recovery) (d) or HU (2mM, 30h). SuperPlots of U-ssDNA foci from n=2 independent experiments are shown (100-200 cells/replicate). Each shape (circle or triangle) represents the average of each replicate; black lines indicate the mean ± SD. **(f)** qRT-PCR of A3B mRNA in U2OS cells transfected with the indicated siRNAs. Data are mean ± SD of n=3 experiments. Statistical significance was determined by un-paired two-tailed Student's *t* test. ***$p \leq 0.001$; **$p \leq 0.01$. **(g)** Western blot of BRCA2 and A3B in U2OS cells transfected with the indicated siRNAs. α-tubulin serves as the loading control. **(h, i)** Quantification of U-ssDNA foci in U2OS cells transfected with indicated siRNAs followed by cisplatin (h) or HU (i) treatment as described above. SuperPlots of mean ± SD, n=2 are described above (e). **(j)** Western blot of BRCA2, GFP-tagged A3A (WT or C106S), or HA-tagged A3B (WT or E255Q) in A3A/A3B DKO cells transfected with indicated siRNAs followed by transfection with indicated plasmids. Vinculin is the loading control. **(k)** Western blot of BRCA2 in U2OS cells transfected with the indicated siRNAs. GAPDH is the loading control. **(l)** DNA fiber assay. Top: IdU/CldU labeling scheme with and without HU treatment. Bottom: Scatter plots of IdU tract lengths in cells transfected with indicated siRNAs +/- HU. 100-200 Fibers were analyzed/experiment. Statistical significance was determined by un-paired two-tailed Student's *t* test. ****$p \leq 0.000$; *$p \leq 0.05$. A second biological replicate is presented in Supplementary Data 2. **(m)** Neutral comet assay. Quantification of average tail moment in U2OS cells transfected with the indicated siRNAs and treated with HU as described above. 100-150 comets/experiment were analyzed. Data are mean ± SD of n=3. Statistical significance was determined by un-paired two-tailed Student's *t* test. ***$p \leq 0.001$; **$p \leq 0.01$. **(n)** Quantification of micronuclei-positive cells in U2OS cells transfected with indicated siRNAs for 48hrs followed by treatment with cisplatin (50µM, 1h; 48h recovery) or HU (2mM, 30h; 24h recovery). Data are mean ± SD of n=3 independent experiments with three technical replicates. 200-250 cells/replicate. Statistical significance was determined as described in (m). ***$p \leq 0.001$; **$p \leq 0.01$; *$p \leq 0.05$. **(o)** Western blot of BRCA2 and A3B in PEO1 cells transfected with the indicated siRNAs. Vinculin is the loading control. **(p, q)** Colony formation assay of PEO1 cells treated with cisplatin (p) or hu (q) for 4 days followed by 6 days of recovery. Error bars represent SD represents a biological replicate (n=2) with technical replicates. Western blot images presented here are representative of three or more western blots with similar results. Source data are provided as a Source Data file.

Supplementary Figure 7

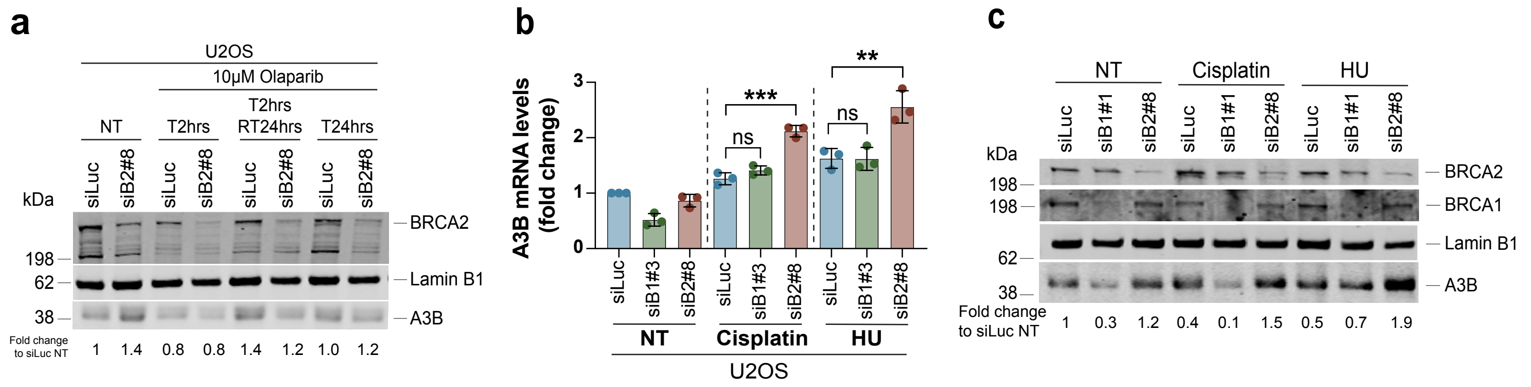

Supplementary Fig. 7. APOBEC33B expression upon replication stress contributes to increased genomic instability in BRCA2-deficient cells.
(a) Western blot analysis of BRCA2 and A3B in U2OS cells transfected with the indicated siRNAs and treated with Olaparib (10µM, 2h; 24h recovery). Lamin B1 is the loading control. One of three experiments with similar results is presented; A3B expression is normalized to loading control prior to fold change compared to siLuc untreated (NT). **(b)** qPT-PCR of A3B mRNA in U2OS cells transfected with the indicated siRNAs followed by treatment with cisplatin (50µM, 1h; 24h recovery) or HU (2mM, 30h). Data are mean ± SD of n=3. Statistical significance was determined by un-paired two-tailed Student's *t* test. ***p≤0.001; **p≤0.01; ns: not significant p>0.05. **(c)** Western blot of BRCA2, BRCA1 and A3B in nuclear extracts of U2OS cells transfected with the indicated siRNAs. Cells were treated as in (a). Lamin B1 serves as the loading control. A3B xpression is normalized to loading control prior to fold change compared to siLuc untreated (NT). One of three experiments with similar results is represented here. Source data are provided as a Source Data file.

Supplementary Figure 8

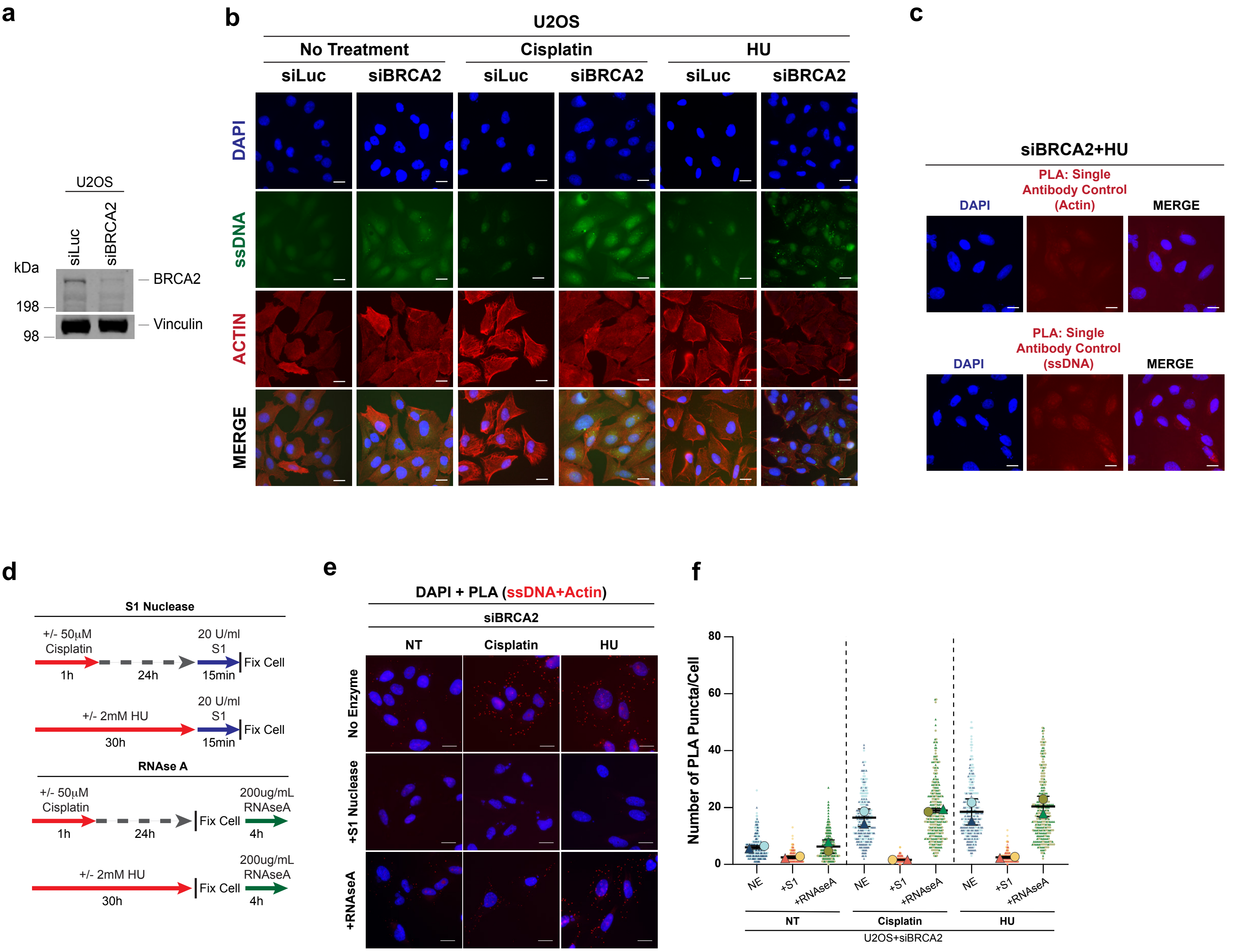

Supplementary Fig. 8: BRCA2 deficiency induces APOBEC3B-, UNG2-, and APE1-dependent accumulation of cytoplasmic ssDNA.

(a) Western blot of BRCA2 in U2OS cells transfected with the indicated siRNAs. Vinculin is the loading control. One of three western blots are shown with similar results are represented. **(b)** Representative images of ssDNA and actin in U2OS cells transfected with the indicated siRNAs and then treatment with cisplatin (50µM, 1h; 48h recovery) or HU (2mM, 30h; 24 h recovery). Scale bar represents 20µm. One of three experiments is represented. **(c)** Representative images of single antibody controls for PLA using anti-ssDNA or anti-actin in siBRCA2 U2OS cells treated with HU (2mM, 30h). **(d)** Schematic of treatment timeline +/- cisplatin or HU, followed by S1 nuclease (20U/mL) or RNAseA (200µg/mL). **(e)** Representative PLA images using anti-ssDNA and anti-actin in U2OS cells transfected with the indicated siRNAs and treated with cisplatin or HU. **(f)** Quantification of PLA puncta per cell from (e). SuperPlots of 2 independent experiments (100-200cells/replicate) are shown. Each highlighted shape (circle or triangle) represents the average of each replicate, with the black lines representing the mean ± SD of n=2 independent experiments. Source data are provided as a Source Data file.

Supplementary Figure 9

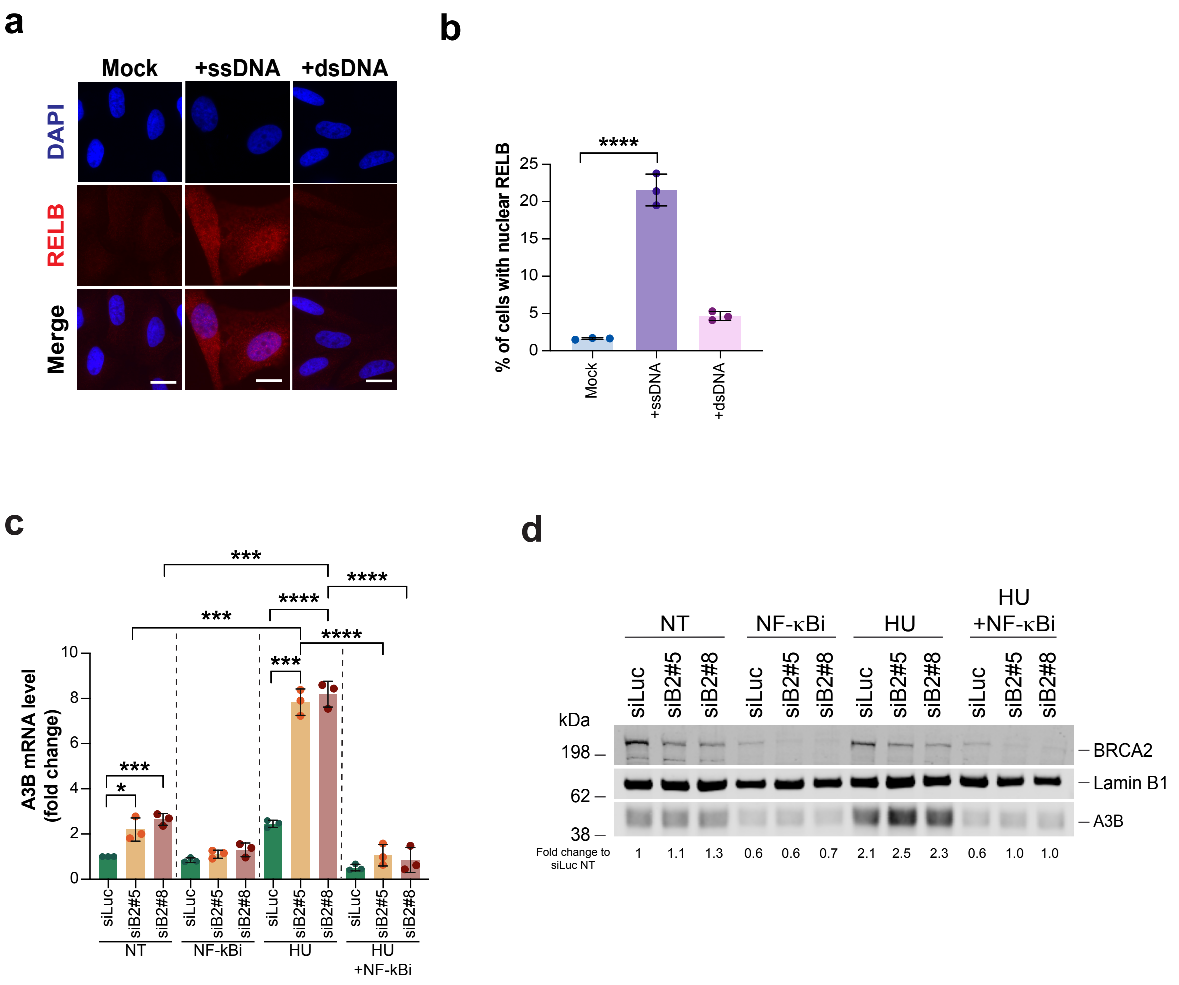

Supplementary Fig. 9: NF-kB signaling drives increased APOBEC3B expression upon replication stress.

(a) Representative images of RELB immunostaining in U2OS cells transfected with mock, ssDNA, or dsDNA for 24h. One of three independent experiments is presented. Scale bar represents 20µm. **(b)** Quantification of RELB positive cells from (a). Data are mean ± SD of n=3 independent experiments. Statistical significance was determined by unpaired, two-tailed Student's *t* test. ****p≤0.0001. **(c)** qRT-PCR of A3B in U2OS cells transfected with the indicated siRNAs and treated with NF-kBi (2h), followed by continued NF-kBi treatment during HU exposure (2mM, 30h). Data are mean ± SD of n=3. Statistical significance was determined by un-paired two-tailed Student's *t* test. ****p≤0.0001; ***p≤0.001; *p≤0.05. **(d)** Western blot of BRCA2 and A3B in U2OS cells transfected with the indicated siRNAs and treated +/- NF-kBi (10µM, 2h) with or without HU (2mM, 30hrs). A3B expression is normalized to loading control prior to fold change compared to siLuc untreated (NT). One of three experiments with similar results is shown. Source data are provided as a Source Data file.

Supplementary Table 1: RNA interference (siRNA) sequences

Targets	Sequences
siLuc	5’-CGUACGCGGAAUACUUCGA-3’
siAPE1#1	5’-CCUGCCACACUCAAGAUCU-3’
siAPE1#2	5’-GAACAAAGACUACUAAUGA-3’
siAPOBEC3A-3B	5’UGACCUACGAUGAAUUUAA-3’
siAPOBEC3B#2	5’-CCUCAGUACCACGCAGAAAUGUGC’3’
siAPOBEC3A#3	5’-ACUUGAUGGAUCCACACAUAUUCA-3’
siBRCA1#1	5’-CAACAUGCCCACAGAUCAA-3’
siBRCA1#3	5′-CAGCUACCCUUCCAUCAUA-3′
siBRCA2#5	5’-GAAACGGACUUGCUAUUUA-3’
siBRCA2#8	5’-UAAGGAACGUCAAGAGAUA-3’
siSMUG1	5’-CCUUUGGGGAAGUAAGCAUGGUCCG-3’
siUNG2#1	5’-GGGACAGGAUCCAUAUCAU-3’
siUNG2#3	5’GCAGUUGUGUCCUGGCUAA-3’

Supplementary Table 2: Antibodies

Target	Species and type	Source (Catalog #)	Type	Dilution
53BP1	Rabbit polyclonal	Bethyl (A300-272A)	Immunofluorescence	IF: 1/2000
Goat anti-Mouse IgG, Alexa Fluor 488	Goat Polyclonal	Thermo Fisher Scientific (A10684)	Fiber Secondary	F: 1/400
Goat anti-Rat IgG, Alexa Fluor 555	Goat Polyclonal	Thermo Fisher Scientific (A21434)	Fiber Secondary	F: 1/400
alpha Tubulin	Mouse monoclonal	Santa Cruz Biotechnology (sc-5286)	Western Blot Primary	WB: 1/3000
APOBEC3B	Rabbit monoclonal	Cell Signaling (41494)	Western Blot and Immunofluorescence Primary	WB: 1/3000; IF:1:500
β-Actin	Rabbit monoclonal	Cell Signaling (4970S)	Immunofluorescence and PLA Primary	IF and PLA: 1/1000
BRCA1 (SD118)	Mouse monoclonal	Calbiochem (OP107)	Western Blot Primary	WB: 1/3000
BRCA2	Rabbit polyclonal	Bethyl (A300-005A)	Western Blot Primary	WB: 1/3000
BrdU (detect CldU)	Rat monoclonal	Abcam (ab6326)	Fiber Primary	F: 1/400
BrdU (detect IdU)	Mouse monoclonal	BD Biosciences(555627)	Fiber Primary	F: 1/500
Cyclin A	Mouse	BD Biosciences(611268)	Immunofluorescence Primary	IF: 1/500
FLAG M2	Mouse monoclonal	Sigma-Aldrich (F1804)	Western Blot and Immunofluorescence Primary	WB: 1/3000; IF: 1/10000
GAPDH	Mouse monoclonal	Biolegend (649202)	Western Blot Primary	WB: 1/3000
GAPDH	Rabbit monoclonal	Invitrogen (MA5-33140)	Western Blot Primary	WB: 1/3000
GFP (GF28R)	Mouse monoclonal	Invitrogen (MA5-15256)	Western Blot Primary	WB: 1/3000
HA	Rabbit polyclonal	Bethyl (A190-108A)	Western Blot Primary	WB: 1/3000
Lamin B1	Rabbit monoclonal	Cell Signaling (#12586)	Western Blot Primary	WB: 1/3000
c-Myc	Rabbit polyclonal	GeneTex (GTX103436)	Western Blot Primary	WB: 1/3000
NF-kB RELB	Rabbit monoclonal	Cell Signaling (10544)	Immunofluorescence Primary	IF: 1/500
PCNA	Rabbit monoclonal	Abcam (ab2426)	Immunofluorescence Primary	IF: 1/400
RELB	Mouse monoclonal	Santa Cruz Biotechnology (sc48366)	Immunofluorescence Primary	IF: 1/500
Ref-1 (APE1)	Mouse monoclonal	Santa Cruz Biotechnology (Sc17774)	Western Blot Primary	WB: 1/3000
Phospho-RPA32 (S33)	Rabbit polyclonal	Bethyl (A300-246A)	Immunofluorescence Primary; Western Blot Primary	IF: 1/2000; WB: 1/3000
ssDNA (clone F7-26)	Mouse monoclonal	Millipore (MAB3299)	and PLA Primary	IF: 1/200; PLA: 1/200
SMUG1	Mouse	Santa Cruz (sc-514343)	Western Blot Primary	WB: 1/3000
UNG	Rabbit polyclonal	Novus (NBP1-49985)	Western Blot Primary	WB: 1/3000
Vinculin	Mouse monoclonal	Invitrogen (MA5-11690)	Western Blot Primary	WB: 1/3000
DAPI		Vector Laboratories (H-1200-10)	Immunofluorescence Mounting Media	
FLAG-ΔUNG-DsRed	Recombinant Protein	PMID: 32956035, Generated in-house	U-DNA Probe	IF: 4ug/ml
IRDye 680RD (anti-Mouse IgG)	Goat	Licor (925-68070)	Western Blot Secondary	WB: 1/10000
IRDye 680RD (anti-Rabbit IgG)	Goat	Licor (925-68071)	Western Blot Secondary	WB: 1/10000
IRDye 800CW (anti-Mouse IgG)	Goat	Licor (925-32210)	Western Blot Secondary	WB: 1/10000
IRDye 800CW (anti-Rabbit IgG)	Goat	Licor (925-32211)	Western Blot Secondary	WB: 1/10000
IRDYE 800CW Streptavidin		Licor (926-32230)	Dot Blot Primary/Detector	WB: 1/10000
Fluorescein (FITC) Anti-Rabbit	Rabbit	JacksonImmunoResearch (111-095-003)	Immunofluorescence Secondary	IF: 1:200
Rhodamine Red-X Anti-Mouse	Mouse	JacksonImmunoResearch (115-295-003)	Immunofluorescence Secondary	IF: 1:400
Rhodamine Red Anti-Rabbit IgG	Rabbit	JacksonImmunoResearch (111-295-003)	Immunofluorescence Secondary	IF: 1:200
Fluorescein (FITC) Anti-Mouse	Mouse	JacksonImmunoResearch (115-097-003)	I Immunofluorescence Secondary	IF: 1/200

WB: Western Blotting, IF: Immunofluorescence, PLA: Proximity Ligation Assay, F: Fiber

Supplementary Table 3: qPCR and PCR primer sequences

Primer Name/Target	Sequence	Sequence Source	Synthesis Source
A3B qPCR Forward	5’-GAATCCACAGATCAGAAATCCGA-3’	Abby Green	IDT
A3B qPCR Reverse	5’-TTTCACTTCATAGCACAGCCA-3’	Abby Green	IDT
Human Actin qPCR Forward	5’-CCAACCGCGAGAAGATGA-3’	Oh, S. et al. PMID: 34389714	EtonBioscience
Human Actin qPCR Reverse	5’-CCAGAGGCGTACAGGGATAG-5’	Oh, S. et al. PMID: 34389714	EtonBioscience
Q205	5’- GAAAAGGGACCCATGCACAG-3’	Venkatesan,S. et al. PMID: 33947663	IDT
Q210	5’-TAGAGTCAGGGATGGGGTCA-3’		IDT
siAPE1#2_siRNA Resistance Mutagenesis Forward	5’-TGAACCAAAACCACCAACGAT-3’	This Paper	IDT
siAPE1#2_siRNA Resistance Mutagenesis Reverse	5’- ATCGTTGGTGGTTTTGGTTCA -3’	This Paper	IDT
UNG AMP Forward	5’- CGCTTTCCAAATAGCCTCCAC-3’	This Paper	IDT
UNG AMP Reverse	5’-CTCTTTCTCTGGGCTGGGTC-3’	This Paper	IDT

Supplementary Table 4: Oligonucleotide sequences

Oligonucleotide name	Sequence	Sequence Source	Synthesis Source
FAM tagged 65 nt ssDNA	5'-FAM-CACACCACGTCTACTTGACGTC CTATGTCCGGAATACCGTTTTGGCAC TATCTTGCTAAGGCCATC-3'	This Paper	IDT
65 nt complementary ssDNA	5'-GATGGCCTTAGCAAGATAGTG CCAAAACGGTATTCCGGACATA GGACGTCAAGTAGACGTGGTGTG-3'	This Paper	IDT

Supplementary Table 5: Recombinant DNA

Recombinant DNA	Source
Plasmid: 3xFLAG-ΔUNG	Pálinkás HL et al. 2020<br>PMID: 32956035
Plasmid: UDG Double Nickase	SantaCruz Biotechnology<br>(sc-403189-NIC)
Plasmid: pUNG2_EYFP	Torseth et al. 2012<br>PMID: 22521144
Plasmid: pCDNA3_APOBEC3A_WT - GFP	Gifted by Abby Green
Plasmid: pCDNA3_APOBEC3A_C106S - GFP	Gifted by Abby Green
Plasmid: pCDNA3_APOBEC3B_WT- HA	Byeon et al. 2016 PMID: 27163633
Plasmid: pCDNA3_APOBEC3B_E255Q - HA	Byeon et al. 2016 PMID: 27163633
Plasmid: Plasmid: N-Myc_APE1	Sino Biological (H616161-NM)
Plasmid: N-Myc_APE1_siAPE1#2 Resistant	This Paper
