# Supplementary material for: BRCA2 deficiency and replication stress drive APOBEC3-Mediated genomic instability
Source: Nat Commun. 2025 Oct 29;16:9544. doi: 10.1038/s41467-025-64578-6 (PMC12572151; doi:10.1038/s41467-025-64578-6)
Supplement: Supplementary file 2 — Description of Additional Supplementary Files [file 41467_2025_64578_MOESM2_ESM.pdf]

Description of Additional Supplementary Files

File name: Supplementary Data 1

Description: UNG2 and APE1 drive stalled fork degradation in BRCA2-deficient cells under replication stress. CldU/IdU tract lengths in cells transfected with the indicated siRNAs and treated with and without HU (5mM, 4h). 150-200 fibers were analyzed per experiment. Second biological replicate to supplementary Fig. 4b.

File name: Supplementary Data 2

Description: A3A/A3B drive stalled fork degradation in BRCA2-deficient cells, but not BRCA1-deficient cells under replication stress. CldU/IdU tract lengths in cells transfected with indicated siRNAs +/- HU. 100-200 Fibers were analyzed/experiment. Second biological replicate to supplementary Fig. 6l.

File name: Supplementary Data 3

Description: RELB nuclear translocation correlates with A3B induction in BRCA2-deficient cells under replication stress. Quantified values of A3B and RELB mean intensity/cell in untreated, cisplatin treated, or HU treated cells. Second biological replication to Fig. 9e-h.
