# Supplementary material for: BRCA2 deficiency and replication stress drive APOBEC3-Mediated genomic instability
Source: Nat Commun. 2025 Oct 29;16:9544. doi: 10.1038/s41467-025-64578-6 (PMC12572151; doi:10.1038/s41467-025-64578-6)
Supplement: Supplementary file 6 — Reporting Summary [file 41467_2025_64578_MOESM6_ESM.pdf]

nature portfolio

Corresponding author(s): Shailja Pathania

Last updated by author(s): Sep 5, 2025

Reporting Summary

Nature Portfolio wishes to improve the reproducibility of the work that we publish. This form provides structure for consistency and transparency in reporting. For further information on Nature Portfolio policies, see our Editorial Policies and the Editorial Policy Checklist.

Statistics

For all statistical analyses, confirm that the following items are present in the figure legend, table legend, main text, or Methods section.

n/a	Confirmed	
☐	☒	The exact sample size (*n*) for each experimental group/condition, given as a discrete number and unit of measurement
☐	☒	A statement on whether measurements were taken from distinct samples or whether the same sample was measured repeatedly
☐	☒	The statistical test(s) used AND whether they are one- or two-sided<br>*Only common tests should be described solely by name; describe more complex techniques in the Methods section.*
☒	☐	A description of all covariates tested
☐	☒	A description of any assumptions or corrections, such as tests of normality and adjustment for multiple comparisons
☐	☒	A full description of the statistical parameters including central tendency (e.g. means) or other basic estimates (e.g. regression coefficient) AND variation (e.g. standard deviation) or associated estimates of uncertainty (e.g. confidence intervals)
☐	☒	For null hypothesis testing, the test statistic (e.g. *F*, *t*, *r*) with confidence intervals, effect sizes, degrees of freedom and *P* value noted<br>*Give P values as exact values whenever suitable.*
☒	☐	For Bayesian analysis, information on the choice of priors and Markov chain Monte Carlo settings
☒	☐	For hierarchical and complex designs, identification of the appropriate level for tests and full reporting of outcomes
☒	☐	Estimates of effect sizes (e.g. Cohen's *d*, Pearson's *r*), indicating how they were calculated

Our web collection on statistics for biologists contains articles on many of the points above.

Software and code

Policy information about availability of computer code

Data collection	qRT–PCR was performed using QuantStudio 3 (Applied Biosystems) using ThermoFisher Scientific Connect Platform.<br>All immunofluoresence images were captured using Axio Imager.M2 (Carl Zeiss) equipped with an Axiocam 506 color camera and controlled by Zen software.<br>All western blot images and colony formation imaging was acquired using LiCor Odyssey.<br>All CellTiter-Glo luminescent signal were obtained using BMG LabTech FLUOstar Omega.
Data analysis	Open Comet (ImageJ plug in) was used for comet tail analysis.<br>Image J was used for U-ssDNA foci, mean intensity and PLA puncta analysis.<br>Image Studio Lite 2 was used for for western blot analysis.<br>Prism (Version 10.2.3, GraphPad Prism Software) was used for Student's t-test analysis, generation of graphs and superplots.<br>SPSS (version 29.0) was used for statistical analysis of all superplots.

For manuscripts utilizing custom algorithms or software that are central to the research but not yet described in published literature, software must be made available to editors and reviewers. We strongly encourage code deposition in a community repository (e.g. GitHub). See the Nature Portfolio guidelines for submitting code & software for further information.

Data

Policy information about availability of data

All manuscripts must include a data availability statement. This statement should provide the following information, where applicable:

- Accession codes, unique identifiers, or web links for publicly available datasets
- A description of any restrictions on data availability
- For clinical datasets or third party data, please ensure that the statement adheres to our policy

Requests for cell lines and other reagents described here can be sent to S.P.

Research involving human participants, their data, or biological material

Policy information about studies with human participants or human data. See also policy information about sex, gender (identity/presentation), and sexual orientation and race, ethnicity and racism.

Reporting on sex and gender	N/A
Reporting on race, ethnicity, or other socially relevant groupings	N/A
Population characteristics	N/A
Recruitment	N/A
Ethics oversight	N/A

Note that full information on the approval of the study protocol must also be provided in the manuscript.

Field-specific reporting

Please select the one below that is the best fit for your research. If you are not sure, read the appropriate sections before making your selection.

☒ Life sciences ☐ Behavioural & social sciences ☐ Ecological, evolutionary & environmental sciences

For a reference copy of the document with all sections, see nature.com/documents/nr-reporting-summary-flat.pdf

Life sciences study design

All studies must disclose on these points even when the disclosure is negative.

Sample size	Sample sizes were determined based on preliminary experiments and chosen according to standards in the field. Statistical analysis using Prism (Version 10.2.3, GraphPad Prism Software) or SPSS (version 29.0) was used to calculate statistical significance between biological samples. Individual p-values are indicated in the and source data.
Data exclusions	No data was excluded from analysis.
Replication	A minimal size of n=3 (key experiments, main figures) or n=2 (supplementary figures) independent biological samples was performed and analyzed as indicated in the figure legends. Key experiments were additionally performed in multiple cell lines.
Randomization	No randomization was performed in this study.
Blinding	No blinding was performed in this study.

Reporting for specific materials, systems and methods

We require information from authors about some types of materials, experimental systems and methods used in many studies. Here, indicate whether each material, system or method listed is relevant to your study. If you are not sure if a list item applies to your research, read the appropriate section before selecting a response.

Materials & experimental systems

n/a	Involved in the study
☐	☒ Antibodies
☐	☒ Eukaryotic cell lines
☒	☐ Palaeontology and archaeology
☒	☐ Animals and other organisms
☒	☐ Clinical data
☒	☐ Dual use research of concern
☒	☐ Plants

Methods

n/a	Involved in the study
☒	☐ ChIP-seq
☒	☐ Flow cytometry
☒	☐ MRI-based neuroimaging

Antibodies

Antibodies used

53BP1 Rabbit polyclonal Bethyl (A300-272A) IF: 1/2000
F(ab')2-Goat anti-Mouse IgG, Alexa Fluor 488 Goat Polyclonal Thermo Fisher Scientific (A10684) F: 1/400
Goat anti-Rat IgG (H+L), Alexa Fluor 555 Goat Polyclonal Thermo Fisher Scientific (A21434) F: 1/400
alpha Tubulin Mouse monoclonal Santa Cruz Biotechnology (sc-5286) WB: 1/3000
APOBEC3B Rabbit monoclonal Cell Signaling (41494) WB: 1/3000; IF:1:500
β-Actin Rabbit monoclonal Cell Signaling (4970S) IF and PLA: 1/1000
BRCA1 (SD118) Mouse monoclonal Calbiochem (OP107) WB: 1/3000
BRCA2 Rabbit polyclonal Bethyl (A300-005A) WB: 1/3000
BrdU (detect CldU) Rat monoclonal Abcam (ab6326) F: 1/400
BrdU (detect IdU) Mouse monoclonal BD Biosciences (555627) F: 1/500
Cyclin A Mouse BD Biosciences (611268) IF: 1/500
FLAG M2 Mouse monoclonal Sigma-Aldrich (F1804) WB: 1/3000; IF: 1/10000
GAPDH Mouse monoclonal Biolegend (649202) WB: 1/3000
GAPDH Rabbit monoclonal Invitrogen (MA5-33140) WB: 1/3000
GFP (GF28R) Mouse monoclonal Invitrogen (MA5-15256) WB: 1/3000
HA Rabbit polyclonal Bethyl (A190-108A) WB: 1/3000
Lamin B1 Rabbit monoclonal Cell Signaling (#12586) WB: 1/3000
c-Myc Rabbit polyclonal GeneTex (GTX103436) WB: 1/3000
NF-kB RELB Rabbit monoclonal Cell Signaling (10544) IF: 1/500
PCNA Rabbit monoclonal Abcam (ab2426) IF: 1/400
RELB Mouse monoclonal Santa Cruz Biotechnology (sc-48366) IF: 1/500
Ref-1 (APE1) Mouse monoclonal Santa Cruz Biotechnology (Sc-17774) WB: 1/3000
Phospho-RPA32 (S33) Rabbit polyclonal Bethyl (A300-246A) IF: 1/2000; WB: 1/3000
ssDNA (clone F7-26) Mouse monoclonal Millipore (MAB3299) IF: 1/200; PLA: 1/200
SMUG1 Mouse Santa Cruz (sc-514343) WB: 1/3000
UNG Rabbit polyclonal Novus (NBP1-49985) WB: 1/3000
Vinculin Mouse monoclonal Invitrogen (MA5-11690) WB: 1/3000
DAPI Vector Laboratories (H-1200-10)
FLAG-ΔUNG-DsRed Recombinant Protein PMID: 32956035, Generated in-house IF: 4ug/ml
IRDye 680RD (anti-Mouse IgG) Goat Licor (925-68070) WB: 1/10000
IRDye 680RD (anti-Rabbit IgG) Goat Licor (925-68071) WB: 1/10000
IRDye 800CW (anti-Mouse IgG) Goat Licor (925-32210) WB: 1/10000
IRDye 800CW (anti-Rabbit IgG) Goat Licor (925-32211) WB: 1/10000
IRDYE 800CW Streptavidin Licor (926-32230) WB: 1/10000
Fluorescein (FITC) AffiniPure® Goat Anti-Rabbit Rabbit Jackson ImmunoResearch (catalog# 111-095-003) IF: 1:200
Rhodamine Red™-X (RRX) AffiniPure® Goat Anti-Mouse Mouse Jackson ImmunoResearch (catalog# 115-295-003) IF: 1:400
Rhodamine Red™-X (RRX) AffiniPure® Goat Anti-Rabbit IgG Rabbit Jackson ImmunoResearch (catalog# 111-295-003) IF: 1:200
Fluorescein (FITC) AffiniPure® Fab Fragment Goat Anti-Mouse Mouse Jackson ImmunoResearch (115-097-003) IF: 1/200

WB: Western Blotting, IF: Immunofluorescence, PLA: Proximity Ligation Assay, F: Fiber

Validation

All antibodies, with the exception of the FLAG-ΔUNG-DsRed Recombinant protein, were obtained from commercial sources and validated according to manufacturer's protocols. Antibodies against APOBEC3B, HA, GFP, UNG, and Ref-1 (APE1) was further validated in cells over expressing the corresponding target. Antibodies against APOBEC3B, BRCA1, BRCA2, Ref-1 (APE1), and UNG were also validated in cell lines using siRNA knockdown for the corresponding target. Antibodies against GFP, HA, FLAG, and c-Myc were also validated in over expression experiments using plasmids containing those tags.
FLAG-ΔUNG-DsRed Recombinant protein was validated in PMID: 32956035.

Eukaryotic cell lines

Policy information about cell lines and Sex and Gender in Research

Cell line source(s)

U2OS (catalog #HTB-96) and UWB1.289 (catalog# CRL-2945) were purchased from American Type Culture Collection (ATCC). U2OS wt (clone #1), U2OS A3B KO (clone #38) and U2OS APOBEC3A/3B (clone#29) KO cells were provided by Dr. Charles Swanton (Cancer Evolution and Genome Instability Laboratory, The Francis Crick Institute, London, UK) and generated as described in PMID: 33947663. PEO1, PEOC4, (PMID: 19654294) CAPAN1 and C2-12 (PMID: 18264087) cells were provided by Dr. Sharon Cantor (UMass Chan Medical School, Worcester, MA 01655 USA). U2OS TRIPZ shBRCA2 cells (Horizon Discovery

	Clone ID: V3THS-376145) were provided by Dr. Ryan Jensen (Yale School of Medicine, New Haven, CT 06520 USA). HeLa cell lines containing doxycycline inducible shAPE1 with or without flag-tagged APE1 complementation was provided by Dr. Gianluca Tell (University of Udine, Udine UD, Italy) (PMID: 36310106). HCT116 chr3+ (MMR competent) with or without UGI cells were provided by Dr. Beáta G Vértessy (BME Budapest University of Technology and Economics, Műegyetem Rkp. 3., Budapest, 1111, Hungary) (PMID: 32956035). DLD-1 (Catalog # CCL-221, ATCC) and DLD-1 BRCA2 knock out (Accession# CVCL_HD57) cells were provided by Dr. David Szuts (Institute of Enzymology, Research Centre for Natural Sciences, 1117 Budapest, Hungary). BICR6 was provided by Dr. Abby Green (Washington University School of Medicine, St. Louis, MO, USA) (PMID: 34347354).
Authentication	U2OS, UWB1.289, and DLD-1 were obtained from commercial repositories (ATCC). Gene knock outs were confirmed by Sanger Sequencing or western blot analysis for protein expression. All other lines have been previously reported in literature referenced in Cell line sources.
Mycoplasma contamination	All cell lines were routinely checked for mycoplasma contamination using a Mycoplasma PCR Detection Kit (Applied Biological Materials Inc. catalog# G238) and confirmed negative.
Commonly misidentified lines (See ICLAC register)	No commonly misidentified cell lines were used in the study

Plants

Seed stocks	N/A
Novel plant genotypes	N/A
Authentication	N/A
